# The use of wearable sensor technology to enhance supportive care in hospitalized palliative patients (Support trial): a prospective preliminary pilot study

**DOI:** 10.1186/s12904-025-01794-3

**Published:** 2025-05-31

**Authors:** Philipp Helmer, Jessica Glück, Anastasios Anastasiadis, Florian Rumpf, Sebastian Hottenrott, Bernd E. Winkler, Patrick Meybohm, Peter Kranke, Carmen Roch, Michael Sammeth

**Affiliations:** 1 Department of Anaesthesiology, Intensive Care, Emergency and Pain Medicine, University Hospital Würzburg, Oberdürrbacher Str. 6, 97080 Würzburg, Germany; 2 Interdisciplinary Center for Palliative Medicine, University Hospital Würzburg, Josef-Schneider-Str. 11, 97080 Würzburg, Germany; 3 University Würzburg, Sanderring 2, 97070 Würzburg, Germany; 4 Department of Electrical Engineering and Computer Science, Coburg University of Applied Sciences and Art, Friedrich-Streib-Str. 2, 96450 Coburg, Germany

**Keywords:** Wearables, Continuous monitoring, Vital parameters, End-of-life, Wearable devices, Telehealth, Biopeak, Biobeat, Movesense

## Abstract

**Background:**

Continuous monitoring of vital parameters using wearable devices offers potential benefits in palliative care, such as early detection of clinical deterioration and improving symptom management. However, evidence supporting their feasibility and utility in hospitalized palliative care patients remains scarce.

**Methods:**

This prospective pilot study aimed to evaluate the feasibility of continuous vital sign monitoring in hospitalized palliative care patients using wrist-worn and chest-wall devices. The study was conducted from October 2023 to November 2024 and included hospitalized patients at a university hospital. Eligible participants were required to provide written informed consent. Patients were monitored for up to 30 days or until discharge or death. Data acquisition focused on the quantity and quality of recorded parameters, including heart rate, respiratory rate, and oxygen saturation, as well as advanced hemodynamic variables. Challenges in recruitment, device performance, and data reliability were assessed.

**Results:**

A total of 275 patients were screened, with 263 excluded for not meeting eligibility criteria. Of the nine patients who provided written informed consent, two withdrew consent before study interventions, leaving seven participants. Among these, one patient completed the maximum study duration, three were discharged to outpatient care, and three died during hospitalization. Wrist-worn devices yielded valid data for 61.5% of the monitored time (median: 57.6%; range: 20.1–78.3%), while chest-wall devices achieved 55.2% (median: 62.3%; range: 3.6–100%). Heart rate and respiratory rate showed excellent reliability (> 99% data availability), whereas oxygen saturation exhibited poor performance (45.1%). The interval between the last recorded device measurement and time of death ranged from 0 to 25 min. Recruitment challenges, including strict consent requirements, resulted in premature study termination, as achieving the target sample size of 25 patients was deemed unfeasible.

**Conclusion:**

This pilot study demonstrates the potential of continuous monitoring technologies in palliative care, but inconsistent data availability limits the ability to recommend their routine use at this stage. Despite these challenges, the promising results highlight the need for further studies to improve device reliability and explore the broader applicability of this technology in palliative care settings.

## Introduction

Caring for patients with advanced, life-limiting illnesses represents a significant challenge. Palliative care focuses on providing holistic support to patients with incurable, progressive diseases and their families based on a multidisciplinary approach [1–3]. Thus, the focus is on achieving the best possible Quality of Life (QoL) rather than pursuing curative treatment. Central to this approach is the alleviation of symptoms such as dyspnea and pain, which affects depending on the primary disease around 50% of patients [4, 5]. Effective management of these distressing symptoms is paramount to ensure optimal supportive end of life therapy and terminal care.

Current practice relies primarily on an external assessment to evaluate and treat distressing symptoms [6–9]. This allows for a standardized assessment using scores derived from various verbal and non-verbal patient expressions and vital signs, eliminating the need for the patient to actively communicate. However, the scores are susceptible to examiner-related subjective bias. Objective surrogate parameters could provide valuable support in guiding therapy [10].

Vital signs, such as heart rate, blood pressure, respiratory rate, and oxygen saturation, are well-established surrogate markers of patient health across most medical fields. However, while these parameters are routinely measured in hospital settings with curative therapy regimes, their use in palliative care remains infrequent [11]. This disparity may be attributed to concerns about patient burden, perceived clinical futility, or fears of “medicalizing death” [11]. Traditional methods, such as blood pressure measurements with cuffs, can cause discomfort and are therefore unsuitable for the use in patients nearing the end of life [11, 12]. Furthermore, it has been shown that sporadic and intermittent measurements of vital parameters in palliative care are insufficient to reliably predict clinical deterioration, and therefore their use in palliative medicine is not recommended [13]. In addition, direct medical actions often do not result from these sporadic measurements.

Consequently, healthcare providers often forgo monitoring to prioritize patient comfort, relying instead on clinical observation [12]. Such approach risks missing continuous subtle physiological changes that could serve as early warnings for clinical deterioration including worsening of burdensome symptoms, thereby delaying potentially beneficial supportive care. Integrating continuous vital parameter measurements into routine palliative care therefore could enhance supportive therapy including care planning by enabling a timely symptom detection [11, 14, 15]. It already has been proved that changes in vital signs, including increased heart and respiratory rates, decreased blood pressure and oxygen saturation are associated with impending death [13, 16]. Continuous monitoring compared to sporadic monitoring or external symptom assessment hence could provide real-time insights and early warnings to improve symptom oriented therapy.

Recent advances in wearable, non-invasive monitoring technology offer a promising solution to support palliative care, by enabling the continuous measurement of vital signs, with minimal discomfort to the patient [15, 17–20]. Recent feasibility studies demonstrated that palliative care patients and caregivers were receptive to using wearable devices, highlighting the potential acceptability and practicality of such approach [21, 22]. Although continuous monitoring has shown promise, current evidence remains very limited. Particularly the feasibility of implementing wearable devices in palliative care in dying patients remains unexplored. However, efforts in this direction could be hampered by the fact that wearable systems based on optical measurements, such as the reflective pulse oximetry, require a sufficiently high peripheral perfusion for accurate data acquisition. In palliative care, it is currently unknown how long this perfusion remains sufficient as patients approach death and complimentary data from intensive care settings suggest early reductions in peripheral perfusion during the dying process [23]. To address these shortcomings in present knowledge, we conducted a pilot study that aims to evaluate the functionality and feasibility of a wrist-monitor for continuous vital sign monitoring and furthermore a chest wall sensor for heart rate monitoring in palliative care patients. Our focus was on the following key questions: (1) What is the time interval between the signal loss of the wrist monitor and the documented time of death? (2) Is continuous vital sign monitoring feasible with wrist- and chest-monitors in palliative care patients? (3) How complete are the acquired datasets? By assessing the performance of wearable systems in palliative care, we aim to pave the way to improving early symptom recognition in order to optimize the supportive care.

## Methods

### Study design

The present trial is a prospective, single-center pilot study. The design adhered to the principles outlined in the Declaration of Helsinki as well as all relevant national and European regulations. All devices employed in the study to collect vital parameters are certified medical devices with CE markings and appropriate regulatory approvals. Ethical approval for this study was obtained from the local Ethics Committee of Würzburg (62/23_awb-am). All participants provided written informed consent prior to study inclusion. The study population consisted of patients admitted to the Interdisciplinary Center for Palliative Medicine at the University Hospital Würzburg. This specialized palliative care unit includes six inpatient beds and primarily cares for adults with advanced, incurable diseases.

All Patients receiving inpatient palliative or terminal care at the Interdisciplinary Center for Palliative Medicine at the University Hospital Würzburg, who were at least 18 years of age and provided written informed consent, were eligible for participation. Exclusion criteria comprised prior participation in this study, the inability to provide informed consent by the patients themselves, an inadequate level in understanding the German language, and the suspected non-compliance. Patients with extensive skin alterations, such as deformities, swelling, redness, edema, infections, or injuries in areas relevant for device application, were also excluded. Other exclusion criteria included tattoos in the measurement area of the wrist-monitor, known allergies to metals, plastics, or silicone, underlying conditions associated with persistent tremors, and severe peripheral vascular diseases affecting the hands.

To investigate the feasibility of wrist-worn wearables in palliative care, the primary endpoint of the study was defined as the time interval between the signal loss of the wrist-monitor and the documented time of death, to investigate the functionality of optical measurement systems during the final stage of life with suspected reduction in peripheral perfusion. Secondary endpoints included the assessment of parameters obtained from advanced hemodynamic monitoring, including continuous blood pressure measurements, as well as the evaluation of data quantity and time resolution of the collected parameters by the wrist-worn and chest sensor.

### Study procedure

Patients were screened during admission to the palliative care unit as part of the standard clinical procedures, which included a comprehensive medical interview and physical examination. Patients were included in the study if they were admitted for inpatient palliative care and provided informed consent. Patients with known allergies to components of the sensors or with conditions that significantly interfered with the measurements were excluded. Eligible patients were provided with detailed information about the study, and written informed consent was obtained prior to participation.

Upon obtaining informed consent, each participant was assigned a unique study-specific identification number to ensure pseudonymization of their data. The wrist-monitor and a chest sensor were both applied, and the continuous collection of vital signs was initiated. One reference measurement for heart rate and blood pressure was taken using the standard upper-arm cuff to calibrate the wristmonitor. Additionally, anthropometric data, including sex, age, body height, body weight, wrist circumference, and Fitzpatrick skin type, were collected. Other relevant clinical data, including comorbidities, Charlson Comorbidity Index (CCI), Karnofsky Performance Status (KPS), and the Eastern Cooperative Oncology Group Performance Status (ECOG) were recorded.

Throughout the hospital stay, the wrist-monitor and chest sensor remained in place. Routine daily visits were conducted to replace the device batteries or charge integrated batteries as required. Study participation was terminated once the patient either passed away or was discharged to outpatient care, with a maximum participation period of 30 days. During the final visit, the length of hospital stay, discharge time, or death time were recorded. The care team could not see the monitoring at any time, so standard care was not affected.

### Employed devices

The devices employed in this study included a chest sensor (MoveSense Medical, Movesense, Vantaa, Finland), classified as a Class IIa medical device and a wrist-monitor (Wrist Monitor BB-613WP, Biobeat Technologies Ltd., Petah Tikva, Israel), certified as a Class IIa medical device.

The wrist-worn monitor utilized reflective pulse oximetry for all reported vital sign measurements, complemented by algorithms based on pulse contour analysis. Thus, not only the standard vital signs but also advanced hemodynamic parameters were automatically derived from these data. Data collected by the device were transmitted via Bluetooth to routers installed throughout the ward. Each patient room was equipped with a dedicated router, and additional routers were strategically placed in the corridors to ensure optimal signal coverage across the ward. These routers transmitted data via Wi-Fi to the manufacturer’s proprietary cloud platform, where the recorded values were processed to calculate vital parameters. The calculated vital signs were then available for real-time monitoring with a latency of less than five seconds through a secure web interface. Due to battery limitations, the devices were replaced daily. The highest available sampling rate was preselected for all devices. During each visit, the correct positioning of the wrist-worn monitors was verified, and adherence to the manufacturer’s usage guidelines was ensured.

The chest wall sensor employed in this study was a single-channel electrocardiogram (ECG) device integrated with motion sensors. It was affixed to patients using specialized long-term electrodes designed for up to seven days of continuous use (Durastrip long-term electrode, Nahtlos AG, St. Gallen, Switzerland). These electrodes featured an integrated fluid reservoir to maintain optimal functionality over extended use periods. The chest wall sensor communicated via Bluetooth protocol with a smartphone (Nokia G20) placed in the patient’s room. A custom-designed application (Longrec, Kaasa Solution GmbH, Düsseldorf, Germany) ran on the smartphone to facilitate data acquisition. All recorded data were stored locally on the smartphone and subsequently transferred to a secure external hard drive upon the completion of the individual study period. For the purposes of this study, only ECG data from the chest wall sensors were analyzed.

### Statistical analysis

All statistical analyses were performed using the R platform (v4.2.0). Descriptive statistics were computed, with non-normally distributed parameters presented as medians and interquartile ranges (IQRs). For data visualization, we utilized the ggplot2 package (v3.3.6). Sample size estimation was challenging due to the lack of valid preliminary studies in the field. Based on a priori assumptions, we planned to recruit 25 patients. To assess the cumulative recording times of different parameters, we employed a modified radar chart overlaid with bar plots. Data availability and relative recording times were visualized using standard bar plots. All analyses were descriptive in nature, as the study was not powered for hypothesis testing. This approach aligns with the preliminary and feasibility-focused objectives of the study.

## Results

### Overview of the cohort

**Fig. 1:**
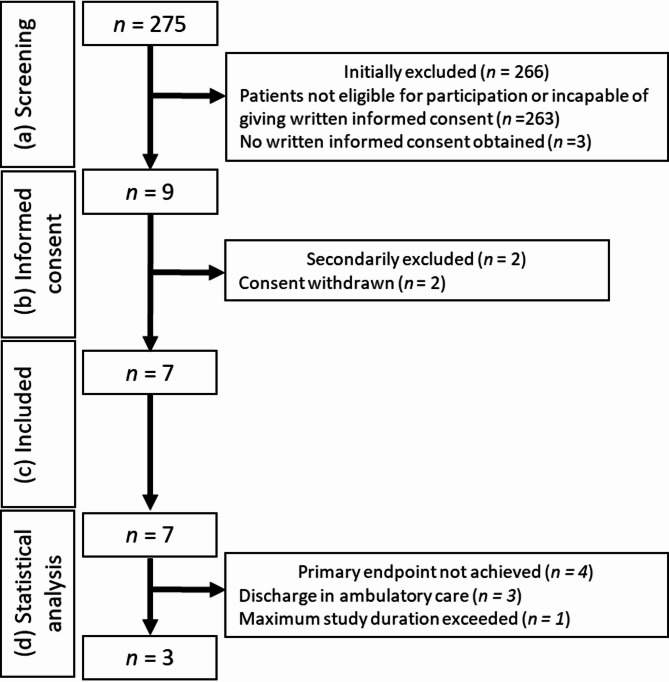
Study flow chart with (**a**) Screening, (**b**) informed consent, (**c**) included patients, (**d**) patients in statistical analysis. On the right side the reasons for exclusion with the corresponding numbers are given

During the study period from October 2023 to November 2024, a total of 275 patients were screened for eligibility (Fig. 1). Of these, 263 patients were initially excluded as they did not meet the eligibility criteria and further three patients were excluded because no written informed consent was obtained. Focusing on the screened patient cohort, 78.2% of the patients died during the hospitalization and 73.5% had an oncological diagnosis. In the end, nine patients provided written informed consent. However, two patients later withdrew their consent and no specific study interventions were performed. Consequently, seven patients were ultimately enrolled and monitored. Among these, one patient completed the maximum study duration of 30 days. Three patients experienced an improvement in their health status and were discharged to outpatient care, while the remaining three patients died during their hospitalization. Following an interim analysis of the study recruitment process, the decision was made to prematurely terminate the study, as achieving the initially targeted sample size of 25 patients was deemed unrealistic.

### Patient characteristics

The cohort included 42.9% female participants (*n* = 3), with the majority (85.7%) exhibiting mild forearm hair (*n* = 6), while one patient had moderate forearm hair. All participants were classified as Fitzpatrick skin type 2. Atrial fibrillation (AF) was present in two patients, while none were diagnosed with obstructive sleep apnea syndrome. Among vascular conditions, two patients had arterial hypertension, and one patients suffered from peripheral arterial disease. An overview of patient characteristics at the time of admission is given in Table 1.

**Table 1:** Patient characteristics for the individual patients and the overall cohort

	Age [y.o.]	Sex	BMI [kg/m^2^]	Height [cm]	Weight [kg]	CCI	ECOG	KPS [%]
Pat 01*	72	W	34.0	168	96.0	8	4	40
Pat 04	70	M	18.5	170	53.4	4	3	40
Pat 05	54	W	21.4	158	53.3	9	3	50
Pat 06*	57	M	20.1	161	52.2	6	3	40
Pat 07	36	W	20.1	164	54.0	6	4	20
Pat 08*	83	M	25.0	158	62.5	7	4	20
Pat 09	79	M	23.6	169	67.5	7	3	40
Median (overall)	70	W 42.9%	21.4	164	54.0	7	3	40
IQR (overall)	55.5; 75.5	NA	20.1; 24.3	158; 169	53.3; 67.5	6; 8	3; 4	20; 40

*Patient died during the study period. y.o. = year old, W = women, M = men, NA = not applicable, BMI = body mass index, CCI = Charlson-comorbidity-Index, ECOG = Eastern Cooperative Oncology Group, IQR = Interquartile range, KPS = Karnofsky Performance StatusQ

### Data quantity analysis of the wrist-worn device

The cumulative device-wearing time —defined as secondary endpoint— totaled 77,133 min across all patients, of which data were successfully recorded for 47,475 min, corresponding to 61.5% of the monitored time. Individual wearing times exhibited substantial variability, with a median of 8,101 min (IQR 1,278.5 min; 12,701 min). Similarly, the percentage of valid data recorded relative to wearing time varied markedly between patients, spanning from 20.1% to 78.3%, with a median of 57.6% (IQR 41.5%; 77.8%).

**Fig. 2:**
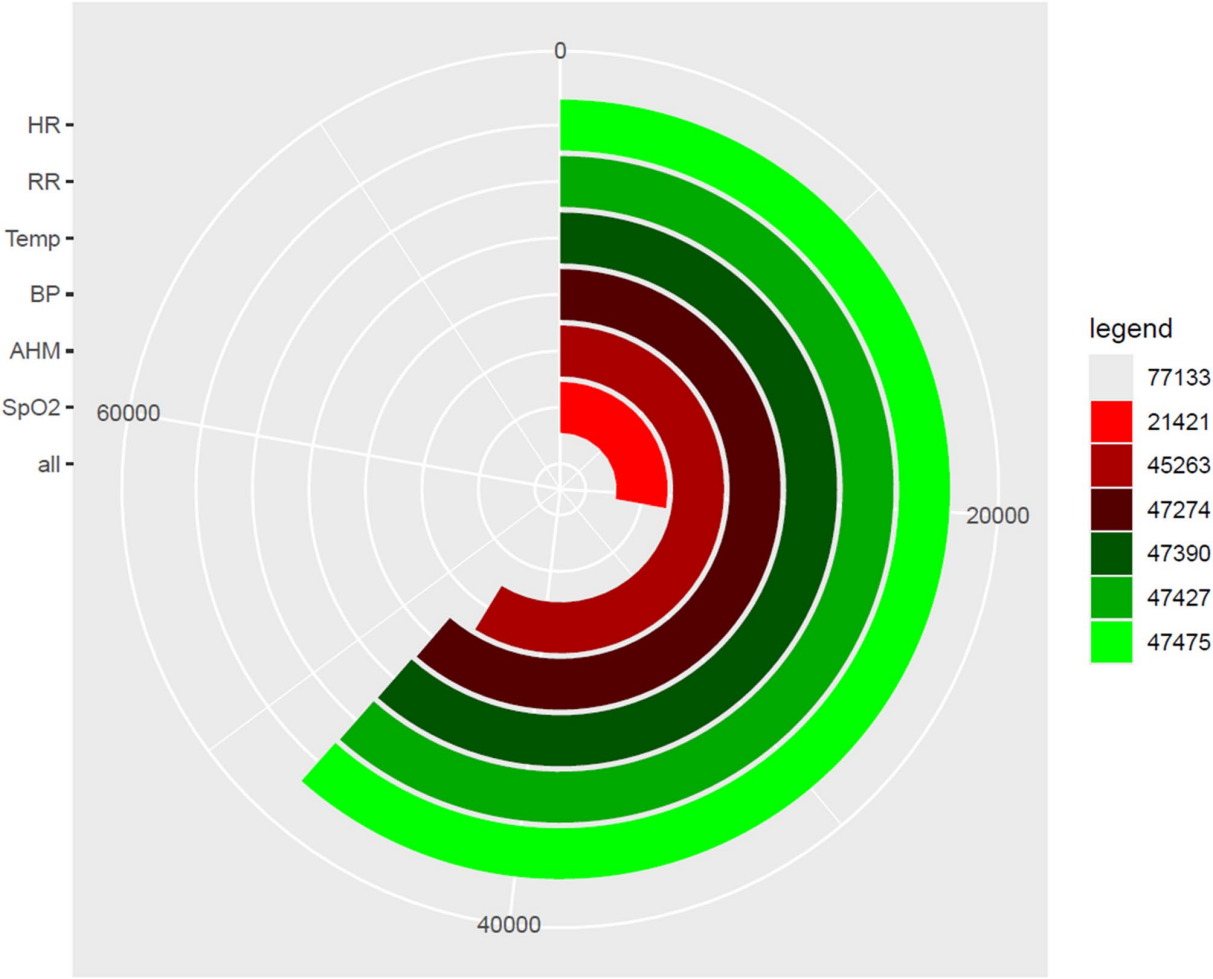
Modified radar chart diagram with bar charts. The different vital parameters are color coded and presented on the left side. The radar chart represents the total using time (77,133 min) with the corresponding total available time spans of the different vital signs. On the right legend the total available time spans of the respective vital signs are given. HR = heart rate, RR = respiratory rate, Temp = temperature, BP = blood pressure, AHM = advanced hemodynamic monitoring, SpO_2_ = peripheral oxygen saturation

The quality of recorded data differed significantly among the measured parameters. Heart rate was captured in 100% of recorded data, followed closely by respiratory rate (99.9%), temperature (99.8%), and systolic and diastolic blood pressure (99.6%). Parameters of advanced hemodynamic monitoring, including stroke volume (SV), cardiac output (CO), systemic vascular resistance (SVR), and cardiac index (CI), were each recorded in 94.8–95.3% of the data. In contrast, oxygen saturation (SpO_2_) exhibited substantially lower performance, with valid data available in only 45.1% of the recordings (Fig. 2).

Dividing the cohort into two subcategories, it can be seen that patients admitted to ambulatory care had higher data quantity (62.4%) compared to patients who died during hospitalization (41.4%).

**Fig. 3:**
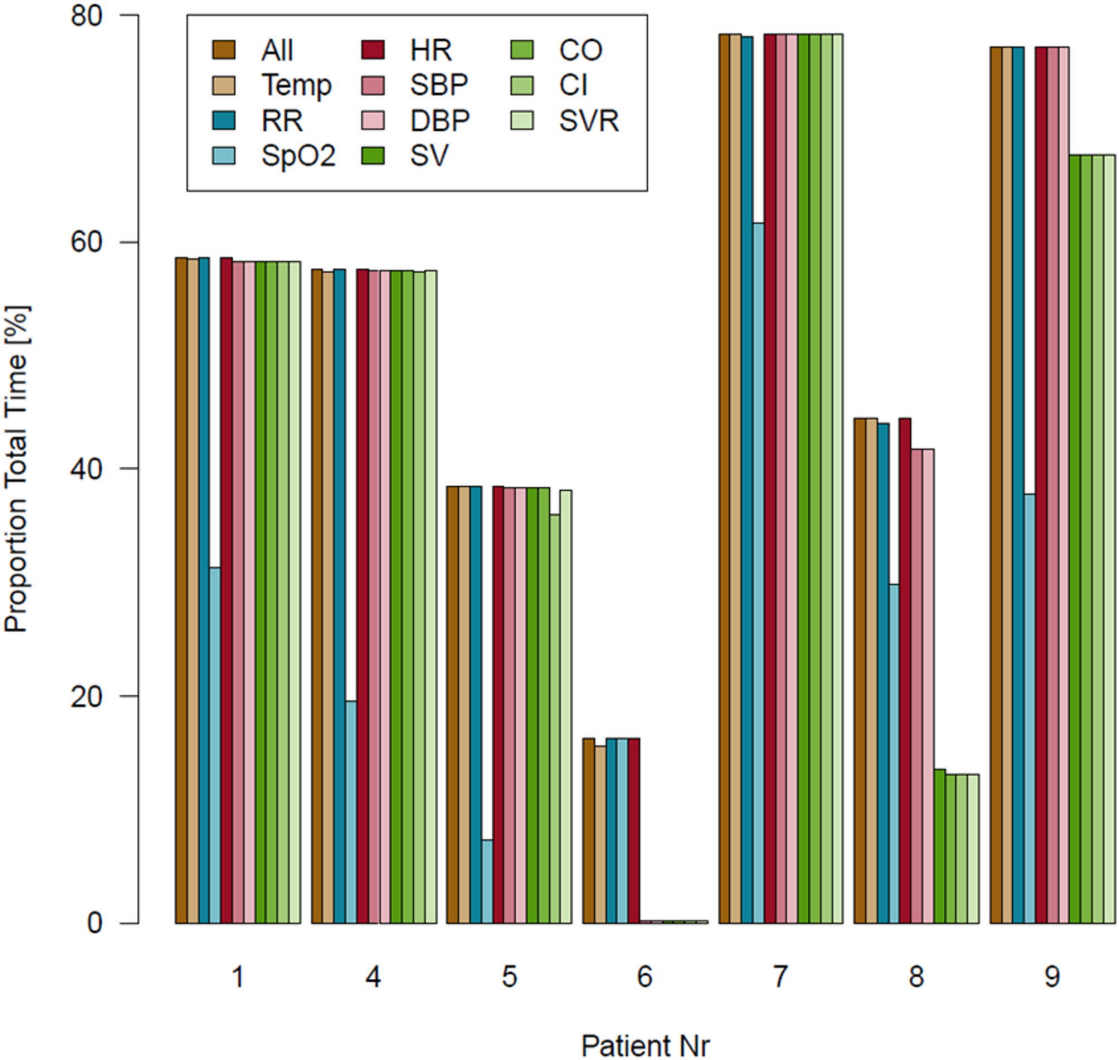
Bar charts showing the respective proportion total time (%) of the successful measured parameters for each patient. The different parameters are color coded. HR = heart rate, CO = cardiac output, Temp = temperature, SBP = systolic blood pressure, CI = cardiac index, RR = respiratory rate, DBP = diastolic blood pressure, SVR = systemic vascular resistance, SpO_2_ = peripheral oxygen saturation, SV = stroke volume

No patient or caregiver removed the devices independently. The data quantity of the various parameters differed strongly across the patients (Fig. 3). Patient 06, who exhibited the poorest data quantity, was the only patient with moderate forearm hair, while all other patients had none or mild forearm hair. Patient 05, with the second-lowest data quantity, showed no identifiable contributing factors, displaying sinus rhythm and mild forearm hair.

The influence of atrial fibrillation (AF) on data quality appeared inconsistent; for instance, Patient 04 with AF demonstrated good data quantity, whereas Patient 08 with AF showed poor data quantity specifically for advanced hemodynamic monitoring parameters. Similarly, peripheral arterial disease (PAD) did not appear to significantly affect data quantity in one patient.

### Data quantity analysis of the chest wall device

**Fig. 4:**
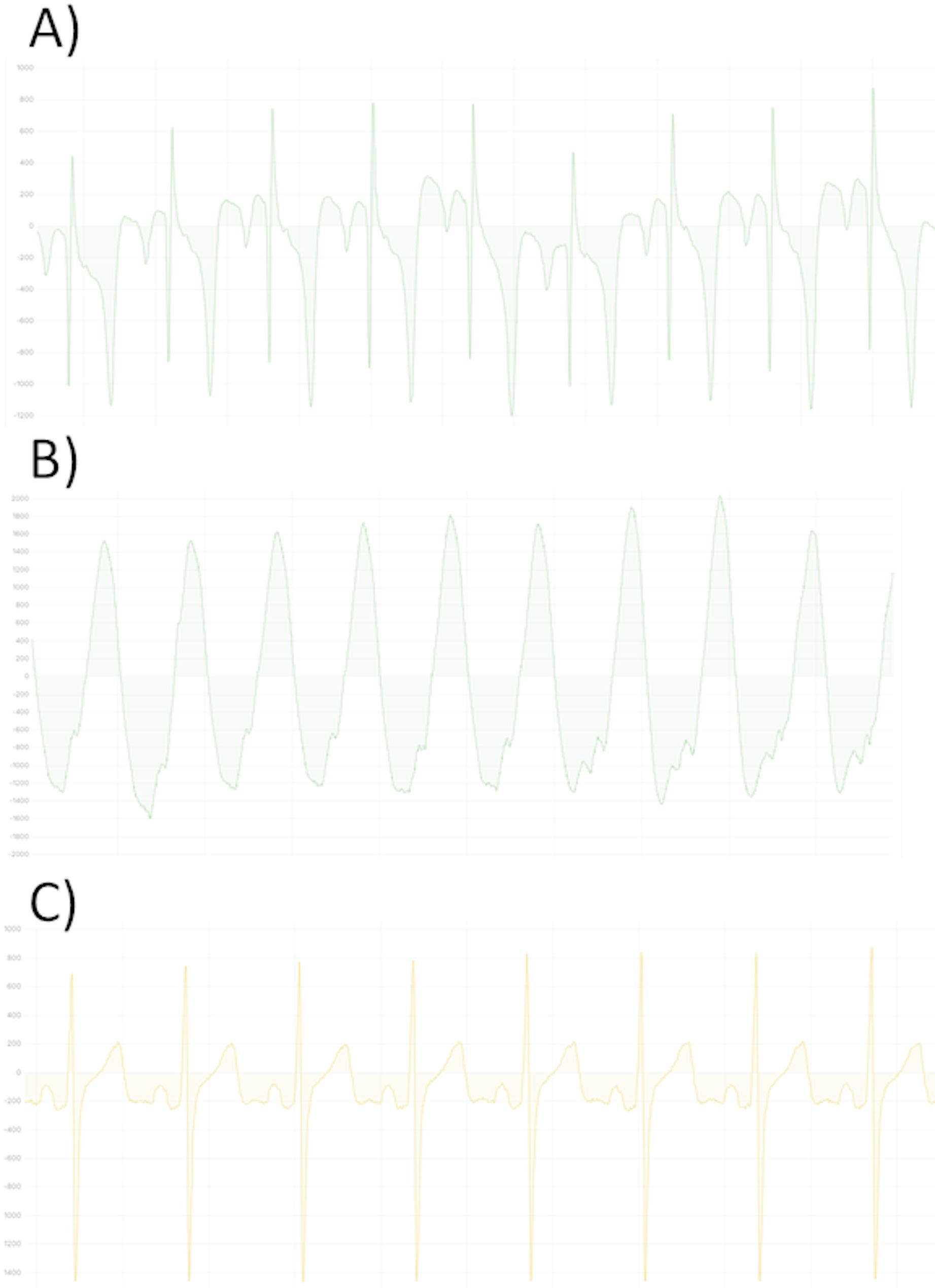
Examples of different ECG recorded with the chest wall sensor. The sampling rate of the ECG was 256 Hz. On the y-axis the signal amplitude is given, depending on the electric current. (**A**) Pathologic ECG of patient 01 with sinus rhythm. (**B**) Ventricular tachycardia of patient 01. (**C**) Sinus Rhythm of patient 06

The cumulative device-wearing time of the chest-wall device was 77,133 min. Of this duration, data were successfully recorded for 42,586 min, corresponding to 55.2% of the monitored time. The median data quantity, expressed as a percentage of the total wearing time, was 62.3% (IQR 44.4%;77.2%), with substantial inter-patient variability ranging from 3.6 to 100%. An example of the recorded one-channel ECG is shown in Fig. 4.

### Functionality of the wrist-worn device during final end-of-life period

Among the nine enrolled patients, three patients died during the study period. The recorded intervals between the last reading of the wrist-worn device and patients death —which constituted the primary endpoint of the study—, varied considerably among the three patients, ranging from 0 min to 25 min. For Patient 01, the documented time of death was 00:50, with the last wrist-worn device measurement at 00:25, resulting in a 25 min gap. Patient 06 passed away at 02:18, with the last recorded measurement from the wrist-worn device at 02:14, resulting in a four minute interval. Patient 08’s documented time of death was 03:50, coinciding precisely with the last measurement from the wrist-worn device.

## Discussion

The present study aimed to evaluate the feasibility of continuous vital parameter monitoring in hospitalized palliative care patients using wearable devices. The overall data availability of the wrist worn device was 61.5% of the total wearing time during the hospitalization period. These findings highlight significant challenges in achieving consistent data acquisition. Among the monitored parameters, SpO_2_ demonstrated the lowest reliability, aligning with prior research that identifies reflective pulse oximetry in wrist-worn devices as prone to interference and high dropout rates [24, 25]. Despite the limited sample size, we successfully determined the time interval between the last measurement recorded by the wrist-worn device and the patients’ death in three cases, as the primary endpoint. This interval was notably brief, ranging from 0 to 25 min.

The measurement gaps of the wrist-worn device are likely to result from the device’s internal algorithm rejecting values of poor signal quality, a phenomenon observed under real-world conditions. The consistent adherence to manufacturer guidelines, including regular device checks and charging, further supports this hypothesis by excluding device failures. Nevertheless, the impact of physiological factors or inherent limitations in the device’s measurement capabilities cannot be fully excluded. It is unlikely that failures are attributed to failures of data transmission, processing or storage errors.

In contrast, the chest-wall ECG device exhibited excellent signal quality despite being a single-lead system with a minimal inter-electrode distance. Due to its compact size and ease of use, the chest-wall sensor does not restrict patients’ mobility, highlighting its significant potential for future applications. Continuous ECG recording without the need for cables enables uninterrupted heart rate monitoring, which could facilitate the early detection of physiological changes and prompt initiation of therapies to alleviate distressing symptoms. Given the excellent quality of the ECG recordings, more advanced analyses are conceivable, although these are beyond the scope of the present manuscript. However, the device’s unapproved data storage software posed significant challenges, including frequent app crashes, unsaved data, and file corruption. These technical failures, causing the missing data, underline the importance of robust software development and validation in the context of continuous monitoring systems.

### Relevance in palliative care

The utility of telehealth interventions and monitoring in palliative care has been supported by numerous studies [26–28], since 2008 [29]. A systematic review of 20 studies concluded that electronic symptom monitoring could improve quality of life (QoL), emotional well-being, and symptom management without increasing costs [30]. On the other hand, more accurate prediction of clinical deterioration or imminent death enables optimization of end-of-life care [16]. Previous research has explored the potential of wearables for home-based monitoring in palliative care. For instance, bed sensors have been employed to track vital parameters in outpatient settings [31]. However, evidence on the clinical utility of continuous monitoring in this context remains mixed [11]. Studies with negative findings have primarily relied on intermittent measurements, typically conducted once daily, which may fail to capture meaningful trends or events.

### Recruitment challenges in palliative care studies

Recruiting participants for clinical trials in vulnerable populations, such as palliative care patients, is notoriously challenging [32–36]. There is great effort to improve the recruitment in studies investigating palliative care settings [36–38]. In randomized controlled trials (RCTs) in this field, only 36.8% of studies meet their planned cohort size based on a priori sample size calculation [39]. The reasons for these challenges remain poorly understood [33], although ethical and methodological complexities likely contribute.

In our study, the primary barrier was identifying eligible patients, who were able to provide written informed consent by themselves. We screened 275 patients and primarily excluded 263 (95.6%) because they did not meet the inclusion criteria, especially the written informed consent criteria. Due to requirements of the responsible ethics committee, patients had to provide informed consent personally, with no option for proxy consent. This significantly limited the pool of potential participants, as many patients at the end of life are either unable to provide written informed consent themselves or have a longer life expectancy because they have only received supportive palliative care as a part of restorative measures or care planning. Future studies should take these study design limitations into account.

### Limitations

This study’s findings are limited by the small sample size, preventing definitive conclusions regarding the primary and secondary endpoints. Consequently, this work should be regarded as a preliminary pilot trial. While the target sample size was not achieved due to recruitment challenges, our preliminary findings provide important insights into the potential and limitations of this approach in the palliative care setting. The study did not include a direct assessment of the devices’ measurement accuracy against established gold standards, which remains a limitation. It should be noted that the daily device replacements caused iatrogenic data interruptions. However, since the devices were only briefly disconnected during the exchange and not throughout the entire charging period, these intervals were not systematically documented. As a result, the actual amount of available data is slightly underestimated. Nevertheless, this effect appears negligible, as the interruptions typically lasted only a few minutes per day.

Vital parameter monitoring is not a “universal” solution in palliative care, as some patients exhibit normal vital signs until death, limiting the utility of continuous monitoring [13, 40]. Additionally, symptoms may arise from the underlying disease that are no longer amenable to causal treatment. Future research should focus on identifying specific patient subgroups that are most likely to benefit from such technologies.

### Future perspectives

This pilot study lays the groundwork for the future application of innovative, non-invasive monitoring technologies in palliative care. These systems have the potential to enhance medical care by providing healthcare teams with continuous insights into patients’ health status, without the limitations of traditional monitoring systems. Many patients in palliative care express a desire to spend as much time as possible at home while feeling secure [41]. Wearable monitoring systems could support this goal by enabling real-time tracking of vital parameters, thereby enhancing safety and facilitating timely interventions.

However, based on our findings, the routine use of wrist-worn devices, employed in this study cannot currently be recommended, particularly given the low reliability of SpO_2_ measurements. Future studies should focus on improving the accuracy and robustness of wearable devices and exploring their applicability in larger, more diverse patient populations. Additionally, chest-wall patches equipped with integrated optical pulse oximetry are now available. Future feasibility studies investigating these devices would be highly valuable, as perfusion at the chest is likely to be superior to that at the wrist, potentially enhancing the accuracy and reliability of measurements. By addressing these challenges, continuous monitoring could become a valuable adjunct to clinical care in palliative settings, offering meaningful benefits to patients and their families.

## Data Availability

The data supporting the findings of this study are available from the corresponding author upon reasonable request and for research purposes only. Data will not be shared for commercial use.

## Abbreviations

AF: Atrial fibrillation
AHM: Advanced hemodynamic monitoring
BMI: Body mass index
CCI: Charlson comorbidity index
CI: Cardiac index
CO: Cardiac output
ECOG: Eastern cooperative oncology group
ECG: Electrocardiogram
KPS: Karnofsky performance status
M: Men
NA: Not applicable
PAD: Peripheral arterial disease
QoL: Quality of life
SV: Stroke volume
SVR: Systemic vascular resistance
SpO_2_: Peripheral oxygen saturation
W: Women
y.o.: Year old
